# Conveyance of texture signals along a rat whisker

**DOI:** 10.1038/s41598-021-92770-3

**Published:** 2021-06-30

**Authors:** Maysam Oladazimi, Thibaut Putelat, Robert Szalai, Kentaro Noda, Isao Shimoyama, Alan Champneys, Cornelius Schwarz

**Affiliations:** 1 Systems Neurophysiology, Werner Reichardt Centre for Integrative Neuroscience, University of Tübingen, Otfried Müller Str. 25, 72076 Tübingen, Germany; 2 Systems Neurophysiology, Hertie Institute for Clinical Brain Research, University of Tübingen, Tübingen, Germany; 3 Department of Engineering Mathematics, University of Bristol, Bristol, BS8 1UB UK; 4 Department of Sustainable Agriculture Sciences, Rothamsted Research, Harpenden, AL5 2JQ UK; 5 Department of Mechano-Informatics, Graduate School of Information Science and Technology, University of Tokyo, Tokyo, Japan; 6 Department of Intelligent Robotics, Toyama Prefectural University, Toyama, Japan; 7 Toyama Prefectural University, Toyama, Japan

**Keywords:** Computational models, Neuroscience, Sensory processing

## Abstract

Neuronal activities underlying a percept are constrained by the physics of sensory signals. In the tactile sense such constraints are frictional stick–slip events, occurring, amongst other vibrotactile features, when tactile sensors are in contact with objects. We reveal new biomechanical phenomena about the transmission of these microNewton forces at the tip of a rat’s whisker, where they occur, to the base where they engage primary afferents. Using high resolution videography and accurate measurement of axial and normal forces at the follicle, we show that the conical and curved rat whisker acts as a sign-converting amplification filter for moment to robustly engage primary afferents. Furthermore, we present a model based on geometrically nonlinear Cosserat rod theory and a friction model that recreates the observed whole-beam whisker dynamics. The model quantifies the relation between kinematics (positions and velocities) and dynamic variables (forces and moments). Thus, only videographic assessment of acceleration is required to estimate forces and moments measured by the primary afferents. Our study highlights how sensory systems deal with complex physical constraints of perceptual targets and sensors.

## Introduction

The study of perception needs to consider the special physical (and chemical) properties of the sensory objects and their interaction with the sensors. In the tactile system, and most prominently in texture discrimination, such physical constraints include friction, arising with moving mechanical contact of integument (hair and skin) and touched object. Frictional phenomena with presumptive high impact on tactile coding are the so-called ‘stick–slip events’ (slips), short jerky movements that originate from storing and releasing energy into elastic object deformations on the microscopic level^1^.

Here, we focus on rats’ vibrissae, or whiskers, tactile hairs that the animals actively move across textures. Rodent vibrissae are tapered giving them extraordinary elasticity^2,3^, and pliability^4–6^. Vibrissae movements vary in characteristic ways^7–9^, such that, in principle, the animal could choose to vary properties of the whisking motion, in order to optimize its performance in a challenging perceptual context. Tracking a point on the vibrissae shaft when rats touched textured surfaces^10,11^ has demonstrated that sequences of frictional stick–slip events can carry a substantial amount of texture information, as well as information about the context, e.g. speed and distance to the texture surface^12^. System identification methods have identified that microslip-like features in the vibrotactile domain are well encoded on the tactile neural pathway^13–19^. Finally, behavioural studies suggest that such stick–slip signals determine perception^20,21^.

From the study of biomechanical properties of whiskers, it has been suggested that dynamical variables, such as force and moment, are likely to be the definitive factors for stimulation of the mechanoreceptors in the follicle^2,4^. Unfortunately, observations of motion of intact whiskers used by a behaving animal are largely limited to whisker kinematics. It is therefore crucial to find out how kinematic variables associated with the whisker’s tip relate to dynamical variables. Moreover, it remains to establish the principles of mechanical transmission of signals along the whisker beam, from the highly pliable tip towards the much stiffer follicle, where neural signals are generated. It would seem that mathematical modelling is necessary to faithfully translate measured whisker kinematics into dynamics.

At present, whisker mechanics has mainly been studied using either linear beam theory^4,22^ or using a quasi-static approximation of a Euler elastica with non-uniform axial properties^23–25^. These approaches are useful for establishing the linear vibration frequencies in contactless whisking, and the quasi-static nonlinear deformations, like buckling, that likely occur when the pliable tip^6^ is in continuous contact with a surface. For example, Goss and Chaouki^25^, using elastic beam theory, worked out criteria for the establishment of tip and line contacts, depending on the distance of the beam to the surface (see also^26,27^). Also, the quasi-static Elastica2D code introduced in^24^ has been successfully used by^28^ to measure the axial follicle force. But none of these techniques can be used to measure the rapid dynamic forces in the transmission of stick–slip waves from the tip to the follicle.

In the spirit of this earlier work, we therefore adapt the elastica formulation to include rapid dynamical deformation while the whisker is in contact with a surface. To do this we employ Cosserat rod-mechanics formulation (e.g.^29^), to model the dynamics of velocity, force and moment at every position along the central axis of the whisker, with complex boundary conditions that can capture the impulsive forces caused by transitions between stick and slip contact. The earlier models likely capture the DC component of the forces at the follicle (which is likely to sense proximity and hardness) whereas our model is able to capture the rapid AC component of these forces, which we argue is how texture is likely to be coded.

In the present work we include the case of tip contact only, but following the approach outlined in^25^ it is straightforward in principle to extend our mathematical formulation to include line contact. In particular, a simple mechanical argument shows that the forces transmitted in the presence of line contact are dynamically equivalent to those of a shorter rod undergoing tip contact.

We combine modelling with a limited number of biomechanical measurements to identify new biomechanical phenomena about how slip-stick transitions at the whisker tip are transmitted to force and moment information at its base. We will show that deflections at the tip, characterized by low moments and large excursions, are rapidly transmitted and result in large moment / short excursion movements at the base, and we will demonstrate that the non-linear modelling can recreate these observed characteristics. Our results strongly suggest that slips generated at the tip are presented in robust fashion to the base, the location of the neurites.

## Results

**Figure 1:**
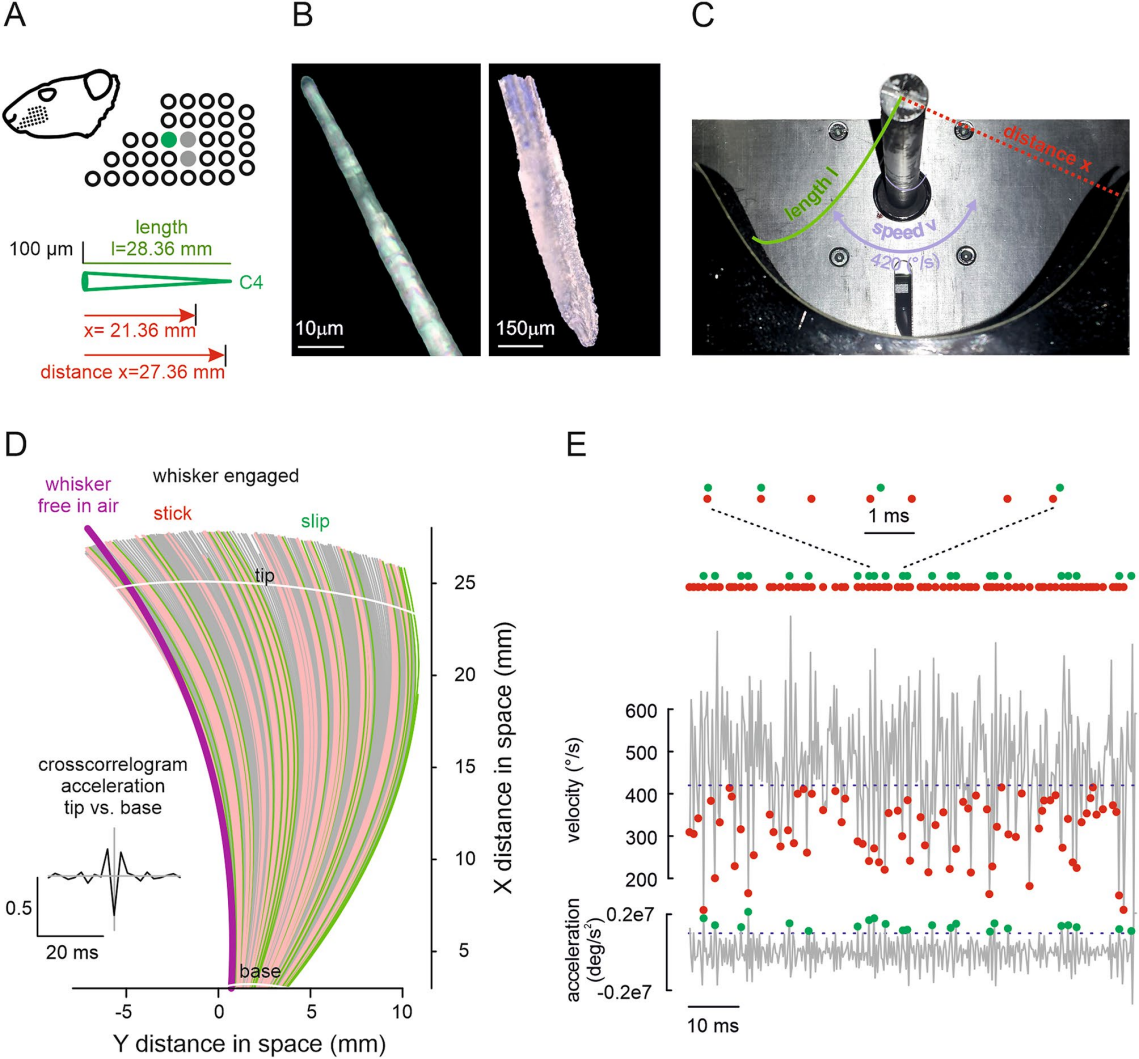
Whisker and biomechanical measurement. (**A**) Rat whisker C4. The rat ‘s head and location of whisker field is shown. The position of C4 is the green dot in the magnified whisker field. Conical shape, length $l$ and distance $x$ (cf. panel C) are shown. (Note that we repeated core measurements using also a C3 and a D3 whisker, the locations of which are indicated by grey dots) (**B**) Microscopic images of whisker tip (left) and base (right). (**C**) Experimental set up (view from the recording camera). The rotating rod is seen on top. At the bottom, the half-cylinder holding the sandpaper is shown. Experimental variables position on whisker (length) $l$, driving speed $v$, and distance $x$ are indicated. (**D**) Stick–slip events in a whisker moving across a texture. Videographic analysis of whisker shape and location in $x$ and $y$ during one protraction (all frames of the video are shown, the sandpaper was located at distance 1 mm less than whisker length: i.e. at $x=27.36$ mm). Red: all frames below the driving velocity—pointing to sticking periods. Green: instances with local maximum of acceleration—pointing to slips. The first frame captured when the whisker was moving free in air (no texture) is shown for comparison (violet). Inset: Cross-correlogram of accelerations at the tip ($x=24$ mm, top white line) vs. that at the base ($x=3$ mm, bottom white line). The grey lines indicate time lag $t=0$ (abscissa) and correlation coefficient $r=0$ (ordinate). (**E**) Method to identify stick and slip events. Sticks (red) were found by thresholding the velocity trace (at driving speed 420°/s) and minimizing the trace below that limit. Slips (green) were found by maximizing the acceleration strips above threshold (2 standard deviations found with movement in air/no contact). On top all events are shown again aligned on the time axis. Further, a short sequence of 4 slips and 7 sticks is shown in the blow-up.

For videography, the follicle end of a plucked whisker (Fig. 1A,B) was glued to a vertical shaft that was made to rotate at constant angular velocity, using a stepper motor (Fig. 1C). The gluing was such as to provide an effective clamped boundary condition, with the whisker free to bend in an approximately horizontal plane. The tip was allowed to contact with the interior of a vertically mounted semi-cylindrical arena, the midpoint of which coincided with the rotation centre of the whisker. We measured the fine-scale, spatio-temporal kinematics of the whisker shaft while the tip was in moving contact with the arena surface clad with sandpapers of different roughness^12^. A rat whisker C4 was used for the experiments. Its length ($l$) was 28.36 mm, and its radius at base was 69 μm. (Core findings of the study were confirmed by measurement of two more whiskers, one C3 and the other D3. Whiskers within one animal or across animals vary in their geometric outline, a variance that we did not attempt to systematically explore with the present experiments). The whisker’s shape was conical with the exception that at the very tip it was truncated at the point where the radius assumed 3 μm. The shaft rotation speed was 420°/s (in some runs also 840°/s and 1260°/s). Two different surface distances were used, $x=l-1$ mm or $x=l-7$ mm. Care was taken to align the intrinsic curvature of the whisker with the horizontal plane and to measure only the movement toward the concave side of the whisker. In the rat’s face such movement would largely correspond to whisker protraction. The movement of the free whisker shaft (i.e. from the parts of the tip not in contact with the sandpaper to the parts close to the follicle that were not fixed and visually obstructed by the glue and rod) was monitored by a camera mounted above operating at ultra-high frame rate (4 kHz if not stated otherwise) (Fig. 1C).

Image processing techniques were used to extract the position of the centreline of the whisker at every frame (resolution in space: 14 µm; in time: 0.25 ms, see “Methods” section). From these data, the normal velocity (in the plane of rotation swept out by the whisker) and acceleration at each point on the shaft were assessed. Kinematic traces of the point 3 mm from the base were used to define ‘sticking phases’ as intervals when the normal velocity reached below the rotational speed of the stepper motor, as well as ‘slipping phases’ as intervals in which whisker acceleration exceeded a value of two standard deviations of the distribution of acceleration measurements during contactless movement (‘movement in air’, as done before in^12^). Colouring the whisker positions according to these sticking and slipping phases reveals prominent and alternating frictional stick–slip events (Fig. 1D), as has been described before^10,11,30^. Minimizing velocity in sticking phases and maximizing acceleration in slipping phases yielded the operationally defined ‘stick and slip events’ (or ‘sticks’ and ‘slips’ for short; green and red dots in Fig. 1E, cf. ^12^). Slip events can be clearly discerned in the acceleration traces of both whisker tip and base, and, confirming an earlier study^12^, they change their appearance when in moving contact with either the P80 or P1200 sandpapers, or when the whisker is engaged with different distances to the texture (Fig. S1). As a note of caution, we wish to emphasize that while the definition of sticks and slips is useful to demonstrate the general relationship of biomechanical variables with stick and slip phases (see below), the precise distinction between slips and sticks in individual cases cannot always be done in straightforward ways, e.g. because in reality there can be complex frictional phenomena like micro-slips and creep-like movements.

### High transmission speed along the whisker

To elucidate the speed of biomechanical transmission from tip to base of the whisker, we plotted the acceleration of tip and base in the plane of whisker movement (tip: $a_{x} (s = 24~\;{\text{mm}})$; base: $a_{x} (s = 3\;{\text{~mm}})$), and calculated their cross-correlation. We found that the acceleration at these two points is negatively correlated, i.e. when the tip speeds up, the base slows down (Fig. 1D, inset). The negative correlation was precise in time: only accelerations at time lag 0 were negative, indicating that conveyance of vibrations are ultra-fast, i.e. non-discriminable using our camera frame rate of 4000 Hz (time bin: 0.25 ms). This finding generalized to all contact conditions studied (i.e. textures, distances, Fig. S1).

### Whiskers vibrate in the second bending mode

**Figure 2:**
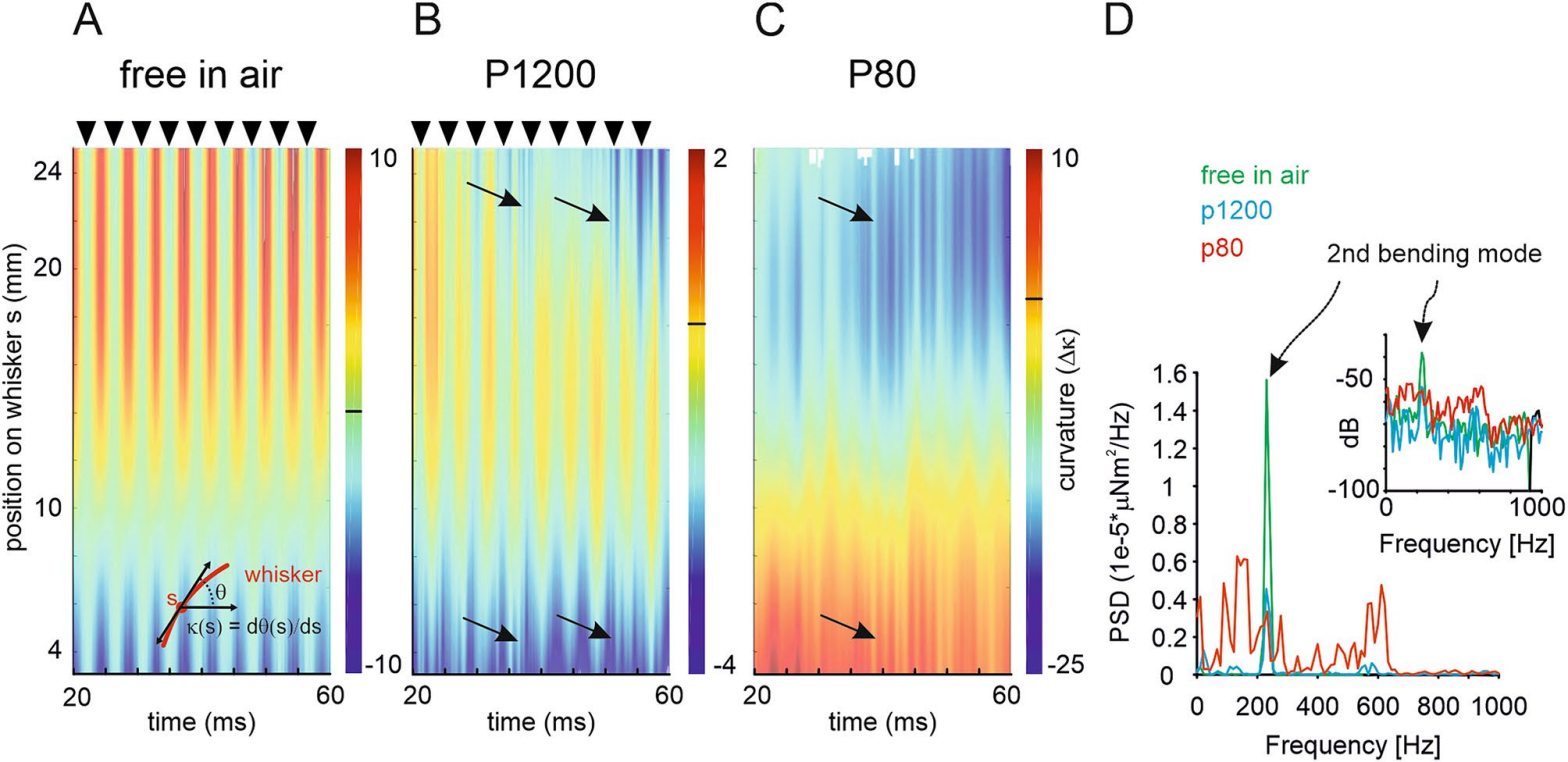
Curvature of the whisker in moving contact. (**A**) Movement in air. Curvature of each point along the beam and across time is colour coded. Inset: Calculation of curvature $\kappa$. The angle $\theta$ at each point of the beam is measured and the curvature calculated as $\kappa \left(s\right)=\theta \text{'}(s)=d\theta /ds.$ We plot $\Delta \kappa$, in which the intrinsic curvature of the whisker (at rest in air) is subtracted. (**B**) Curvature as in A when in contact with a smooth sandpaper (P1200) and (**C**) a rough sandpaper (P80). For both the distance was (distance $x=l-1$ mm; speed $v=420$°/s). Arrows point to curvature changes evoked by stick–slip events being transmitted rapidly along the beam and therefore appearing as vertical stripes. (**D**) Spectra of base movements shown in ABC. Inset shows the same data rescaled to dB. Note the prominent peak in the spectrum at ~ 200 Hz in the ‘free in air’ condition, indicating the 2nd bending mode.

Next, we wanted to find out how the observed opposing movement directions near the tip and the base of the whisker plays out along the entire whisker beam. To that end, we estimated the local curvature (at all pixels on the whisker centreline) by determining the angle $\theta$ spanned by the vector orthogonal to whisker base and the tangential force acting on the whisker beam (Fig. 2A inset). Curvature $\kappa$ is defined by the spatial derivative of $\theta$ ($\kappa (s) = ~d\theta$/$ds$). We plot curvature $\Delta \kappa ={\kappa }_{i}-\kappa$ (i.e. curvature relative to the intrinsic curvature ${\kappa }_{i}$ of the whisker), at all points along the shaft at each sampled time point during a single sweep (Fig. 2A–C). When moving in air (with the sandpaper-coated screen removed) the whisker oscillated between roughly its intrinsic shape ($\Delta \kappa~ = ~0$; arrow heads in Fig. 2A), and a deflection with the tip curving backward ($\Delta \kappa (tip){{~ > ~}}0$) and the base curving forward $( \Delta \kappa (base)~ < ~0$; between arrow heads in Fig. 2A), corresponding to an oscillation in the second bending mode. The result of this vibration mode is that the change in curvature relative to the intrinsic shape changes along the beam, with a node located at about 8 mm distance from the follicle. The oscillation frequency was close to 200 Hz (Fig. 2A).

When the whisker engaged with the smoother sandpaper (grain size P1200; at a distance 1 mm less than its length), $\Delta \kappa$ at the tip changes to forward direction ($\Delta \kappa (tip)~ < ~0$) as expected (Fig. 2B,C). Thus, the free vibrations of the whisker were damped, but they were nevertheless still visible at about the same frequency (arrow heads, Fig. 2B). In addition, rapid waves corresponding to the onset of a slip, became visible. These are seen as the irregular vertical stripes, two of which are marked by arrows. The vertical nature of these stripes again suggests their ultra-fast transmission along the beam. Similar results were observed with P80, the rougher sandpaper (Fig. 2C). The second bending mode was robust against variations of texture engagement. With stronger engagement, either by increasing roughness (Fig. 2C) or decreasing distance (Fig. S1) the node shifted only slightly toward the base (to around 5 mm from the base).

In summary, we note that second order bending with opposite curvature at the tip and base with a node at a distance of 5–8 mm from the base are a characteristic feature of whisker vibrations in varying contact situations. The spectra of whisker vibration when engaged at distance $l-1$ mm are shown in Fig. 2D. The stable second harmonic of the oscillation stands out with movement in air (green) and against the smooth P1200 sandpaper (blue). The fundamental frequency was nearly absent. This oscillatory pattern breaks down when the whisker is engaged with the rough P80 sandpaper (red) because the signal from the slip and stick events begins to dominate the signal.

### Weak and wide tip excursions are transformed into strong and short ones at the base

**Figure 3:**
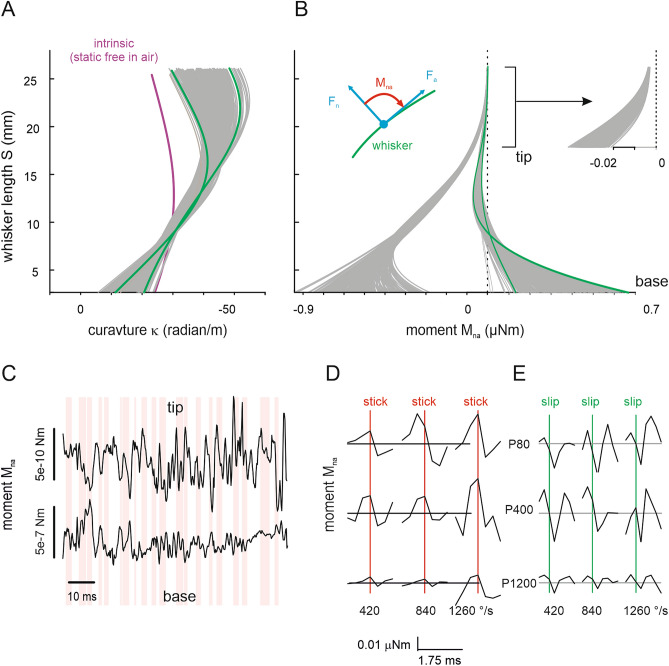
Moment amplification during transmission from whisker tip to base. (**A**) Curvature of whisker in contact with P80 (speed 420°/s). Each line represents one time bin (0.25 ms). Two frames toward the extremes of the curvature are coloured to demonstrate a node of vibration at around 10 mm from the tip (2nd bending mode). (**B**) Moment ${M}_{na}$ as calculated from Eq. (2). Left Inset: Schematic of normal and axial forces ($F_{n} ,$F_﻿a_) and moment (${M}_{na}$) acting in the plane of whisker movement. Moments are negligible at the tip and small negative moment builds up a short distance from the tip. (cf. right inset). The left bundle of lines indicate moment calculated using $\kappa$ (instead $\kappa -{\kappa }_{i}$, cf. Equation 2). Two arbitrary frames are highlighted in green to demonstrate the node of vibration. (**C**) Moment ${M}_{na}$ at tip (s = 24 mm) and base (s = 3 mm) of the whisker (note the three orders of magnitude difference in scale). The red areas indicate periods of sticking ($v<420$°/s). (**D**, **E**) Average moment ${M}_{na}$ with respect to stick (red) and slip (green) events (as identified using the method in Fig. 1B). Nine correlograms taken from traces measured with three different sandpapers and using three driving speeds are shown.

The curvature measurements provide a basis to infer the moment ${M}_{na}(s,t)$ (spanned by the normal force F_n_ and the axial force F_a_ see inset in Fig. 3B) about the vertical axis at every point along the whisker along its centreline $s$ and time $t$. The effect of ${M}_{na}$ is to locally bend the whisker beam at the point in question within its plane of movement. At each point position $s,$ ${M}_{na}$ can be calculated, from the beam’s Young’s modulus ($E$), its measured curvature $\kappa (s$), intrinsic curvature of the whisker ${\kappa }_{i}(s$) (Fig. 3A), and the second moment of area of its circular cross-section

1 $$I\left( s \right) = \frac{\pi }{4}r(s)^{4}$$

**Figure 4:**
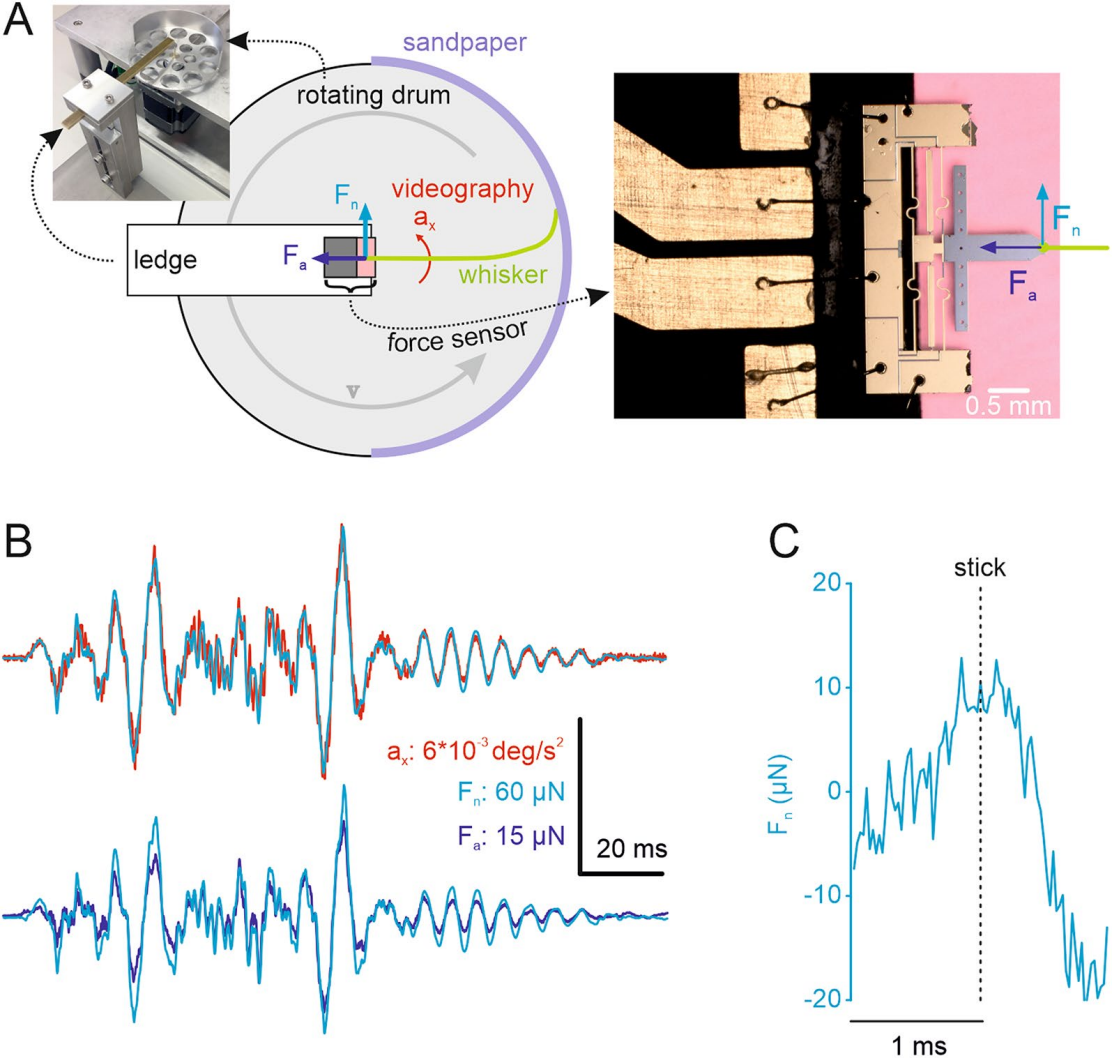
Micro-force measurement at the whisker base. (**A**) Left inset: Photograph of the measurement set-up. The sensor was mounted on an immobile ledge directly above the rotation axis of a rotating drum. The drum was perforated to save weight and contained a wall to fix the sandpaper. Centre: Schematic of measurement set-up. The whisker (green) was mounted on the sensor (dark grey and pink), and brought in contact with the sandpaper (violet), which in turn was rotated by the drum (grey arrow). Two measurements were performed. First, the forces in the plane of whisker movement ($F_{n} ,$F_﻿a_) were directly measured by the piezoresistive sensor (right inset). Second, the lateral acceleration at the base (s = 3 mm) (${a}_{x}$) was assessed by videography (cf. Fig. S1). (**B**) The top graph plots ${a}_{x}$ (red) on top of ${F}_{n}$, the bottom one plots ${F}_{n}$ and ${F}_{a}$. Acceleration is in excellent correspondence to the forces acting on the base. (**C**) Average normal force triggered on stick events. ${F}_{n}$ builds up during sticks (as well as ${F}_{a}$, not shown). Compare with the parallel increment of moment ${M}_{na}$ as calculated from curvature measurements (Fig. 3D).

Assuming a linearly elastic constitutive model for a conical rod with circular cross-section, we then write

2 $$M_{{na}} \left( {s,t} \right) = EI\left( s \right)\left[ {\kappa \left( {s,t} \right) - \kappa _{i} \left( s \right)} \right],$$

(see also Eq. 6 and related text in materials and methods). Combining Eqs. (1) and (2), given the highly tapered nature of the whisker, we note there will be a continuous build up in strength of moment along the whisker as we approach the base end. Thus, tiny forces deflecting the distal pliable part of the whisker lead to a build-up of moment along the whisker beam. This guarantees that the base of the whisker signals a robust moment signal in response to slight tip deflections (Fig. 3B). To quantify the amplification of moment we calculated the ratio of variances of moments measured at the site 1 mm distance from the tip and same distance from the base of the whisker ($\frac{{var}_{tip}}{{var}_{base}}$). This ratio was 1.4e−4 for the C4 whisker shown in Fig. 3B. We studied two more whiskers (one C3 and one D3). These were measured with the drum rotating (cf. Fig. 4) instead of rotating whisker, and analysed in exactly the same way. They yielded still smaller ratios, i.e. higher amplification (C3, length 44.56 mm, ratio: 2.6e−4; D3, length 45.22 mm, ratio: 2.57e−4). Interestingly, the intrinsic curvature ${\kappa }_{i}$ acts to shift the range of moments experienced by the follicle and attached neurites such that a sign change results, a fact which likely contributes to the phenomenon that the whisker vibrates mainly in its second bending mode (cf. Fig. 2 and S1, S2). The time series of M_na_ at tip and base and their inverse relationship can be appreciated in Fig. 3C. Triggering moment at the base by stick and slip events revealed a systematic relationship between the two variables: on average, moment builds up during stick (Fig. 3D) and is released during slip (Fig. 3E).

In summary these results suggest that the taper of the whisker together with its intrinsic curvature play an important role in determining the range of moments experienced in the follicle, the site of neurites of primary afferents, and that stick–slip events feature prominently in the robust moment signal that is received there.

### Acceleration is a proxy for whisker-beam forces and moments

In order to verify the calculations of moment from kinematic data we chose to directly measure the force acting at the whisker base when in contact with the sandpaper. To this end we used a piezoresistive force sensor probe that is capable to measure multiaxial forces in the nano- to microNewton range (Fig. 4A; Fig. S3^31^). The whisker was glued to the probe’s cantilever and the sandpaper was brought into contact with the whisker tip and moved past it using a rotating drum. In this configuration, the force sensor was able to measure the normal (F_n_) and axial force (F_a_) acting at the whisker base (light and dark blue arrows in Fig. 4A). At the same time, we used ultra-fast videography (at a 9.6 kHz frame rate) as described above to measure the acceleration close to the base (${a}_{x}$, red in Fig. 4). We find that F_n,_ F_a_, and a_x_, are strongly correlated ($\left| {r_{{F_{n} }} ,_{{a_{x} }} } \right| = 0.9349;\;\left| {r_{{F_{a} }} ,_{{a_{x} }} } \right| = 0.9302;\left| {r_{{F_{n} }} ,_{{F_{a} }} } \right| = 0.9657$; Fig. 4B). In consequence, the average force observed during sticks shows a clear peak, as exemplified by event-triggered average of F_n_ plotted in Fig. 4C.

In summary, the time series of acceleration measured close to the base provides a reasonable approximation to the time series of forces acting on the base. However, we wish to emphasize that the usage of acceleration as a proxy for forces at the base suffers some limitation because moment (and possibly also forces) are subject to a large augmentation from tip to base while acceleration is not (Fig. S1).

### Cosserat mechanics with friction model recreates1 whisker-beam dynamics

It has been shown that whisker bending in air can be estimated quite well by models based on linear beam theory^4,23^. However, the conical whisker’s highly pliable tip together with frictional forces acting at it renders it highly unlikely that linear beam theory will be sufficient. Rather, it is clear that a formulation is required that allows arbitrarily large, geometrically exact deformations. One way to see this is to calculate the critical Euler load $P_{E}$ defined by

3 $$P_{{E~}} = \frac{{\pi ^{2} EI}}{{4l^{2} }},$$

with $E$ being the Young Modulus, $I$ the second moment of area (Eq. 1), and $l$ the rod length. Applying reasonable assumptions about material constants of a homogenous, isotropic conical rod, this equation yields critical Euler loads in the order of 1 nN. Buckling of the beam is thus expected with minimal forces—widely exceeding those we have measured to be acting at the whisker’s tip. We therefore turned to Cosserat mechanics, which is able to account for gross geometrical non-linearities (see e.g.^29^). Cosserat models capture the dynamics of the space-centroid line (the line intersecting the centre of all circular cross-section areas of the conical rod; Fig. S4A) using a set of coupled partial differential equations in space and time. We deem such spatio-temporal coupling essential to recreate rapidly propagating and highly fluctuating frictional movements at the whisker tip. Such motion could not be captured by a quasi-static Euler–Bernoulli formulation, in which the temporal dimension is neglected. It is noteworthy that the whisker used here has an aspect ratio (length $l$ to cross-sectional radius $r$) of more than 400. For such slender objects it is widely accepted that the effect of shear along the rod’s longitudinal axis is negligible and can be ignored. In this case, a simpler mathematical formulation can be used, which assumes that cross-section areas are normal to the tangent of the space-centroid line (see materials and methods for details).

The whisker is modelled as a truncated cone with length $l$ (from base to truncation point, the latter is called ‘tip’ throughout the paper), and homogeneous linearly elastic material properties (i.e. a constant Young Modulus $E$). The elasticity of the rod for bending movement perpendicular to its longitudinal axis will increase significantly from base to tip, because the circular cross section decreases (see eqns. (1) and (2)). The central variable to describe the whisker mechanics is the local curvature $\kappa \left(s,t\right)$ defined as the first derivative of the local curvature angle $\partial \theta (s,t)/\partial s$. We suppose that the rod has an intrinsic curvature profile ${\kappa }_{i}\left(s\right)$ , which we obtained from measurements of the real whisker. As in the experiments, we assume that the rod moves in the horizontal plane laid out by the intrinsic curvature (with tip bent in the direction of sandpaper movement, Fig. S4A).

Accurate modelling the effects of high-speed dynamical friction between two dry surfaces is a current topic of active research within the field of tribology, with no consensus emerging on which, if any, closed-form low-order model is able to capture the dynamical consequences of surface roughness (see e.g.^32^). We have chosen to use a so-called rate-and-state frictional law (see^33–35^). This approach models the dynamical friction coefficient $\mu$ and resulting friction force ${F}_{T}=\mu p$ (Fig. S4) based on parameters of past sliding history (the interface ‘state’), and the relative velocity between the contacts (the ‘rate’ of deformation, see^34^). This formulation is richer than classical Coulomb friction as it additionally allows setting a parameter $L$ called ‘slip length’, sometimes considered a proxy for roughness. Many geometric and material property parameters were taken as measured. A full list of model parameters is given in Table 1.

**Table 1:** List of all input parameters of the Cosserat and the friction model.

Parameter (measured)	Symbol	Units	Value
Whisker length	$l={s}_{max}$	mm	28.36
Base radius	${r}_{0}$	μm	69
Tip radius	${r}_{1}$	μm	2.5
Intrinsic curvature	${\kappa }_{i}$	rad/m	0
Whisker mass	${m}_{w}$	mg	0.19
2nd mode natural frequency	${\omega }_{2}/(2\pi )$	Hz	230
Truncation length	${l}_{c}$	mm	1.066
Density	$\rho$	kg m^-3^	1295.1
Young modulus	$E$	GPa	3.3
Sound speed	${c}_{s}$	m s^-1^	1596.3
*Parameter (fixed)*			
Driving plate location	$\stackrel{-}{y}/l$	$\varnothing$	0.98
Damping constant	$\delta = 0.5~\alpha \rho c_{s} r_{0}^{3}$	kg m s^-1^	$3.396 \times 10^{{ - 7}} ~\alpha$
Dimensionless damping constant	$\alpha$	$\varnothing$	0.1–1
Friction velocity variation strength	$a$	$\varnothing$	0.035
Interfacial state variation strength	$b$	$\varnothing$	0.049
Friction velocity reference	${V}_{*}$	μm s^-1^	1
*Parameter (varied)*			
Memory length	$L$	μm	5–500

Modelling details of frictional movements of whiskers in contact with specific surfaces will need future versions of the frictional model including also material properties and asperity size and distributions of surface materials. Here we content ourselves with varying $L$ to adjust model behaviour qualitatively to match our principal experimental findings. In particular we asked whether stick–slip can be captured with our approach, and how they are represented by kinematic and dynamical model outputs. We further aimed at capturing the experimentally observed generation of increasing moments along the whisker beam, second bending mode and the rapid transmission of slip events along the beam.

### Model dynamics

**Figure 5:**
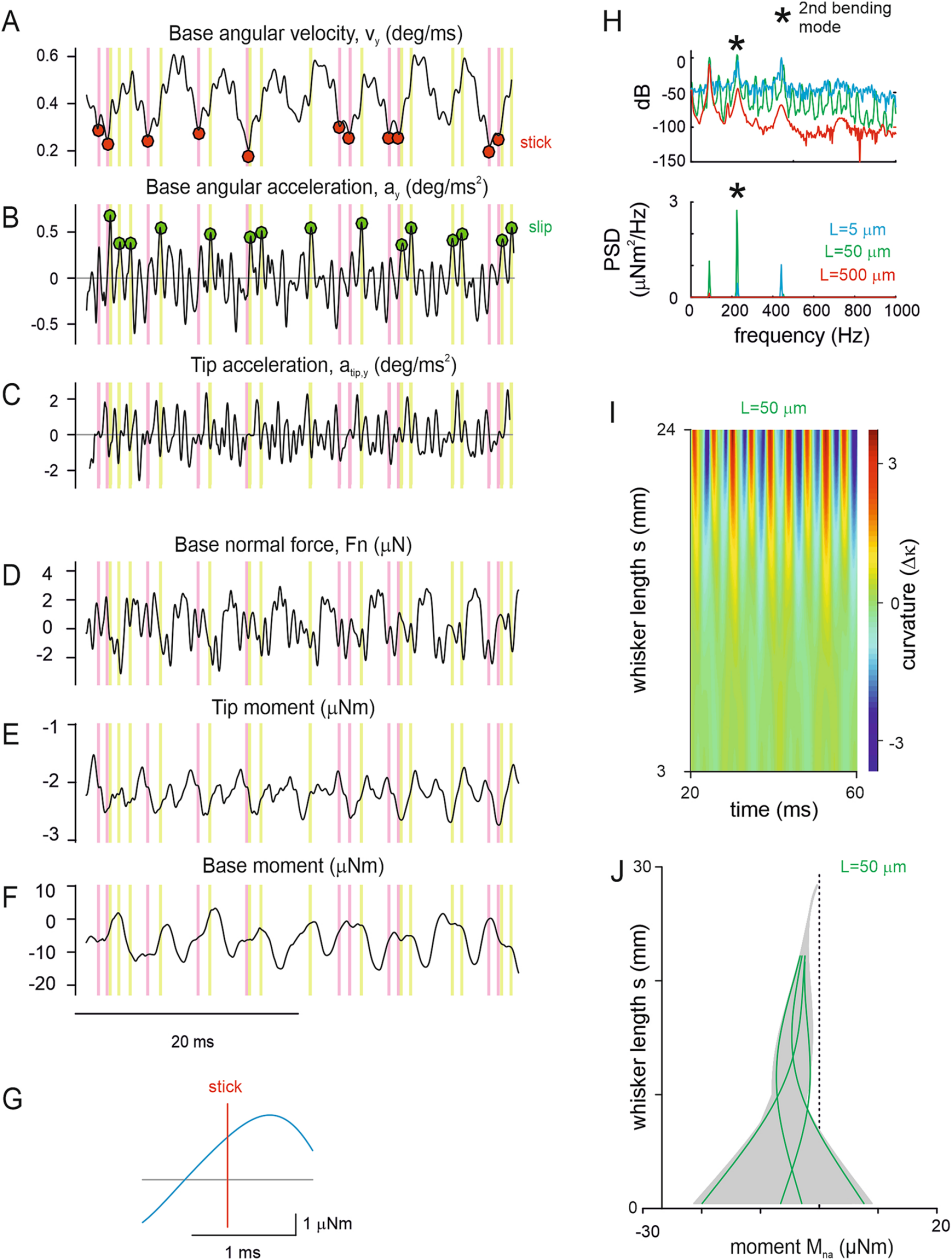
Kinematic and dynamical output variables of the model (see full set of output variables in Table 2). Stick and slip events were identified as in the videographic measurements (cf. Fig. 1E, dots in base kinematic traces in which the identification was done, and lines throughout, red: sticks; green: slips) (**A**–**C**) Kinematic variables (**D**–**F**) Dynamical variables. (**G**) The average normal force to the whisker base builds up to a maximum around a stick event. (**H**) Spectra of moments for model runs using different values of $L$ [5,50,500]. Top and bottom are the same data plotted as dB or PSD. Medium values of $L$ match the experimental data (cf. Fig. 2D) best, as this model best recreates the dominance of the 2nd bending mode (2nd harmonic is marked by an asterisk). (**I**) Curvature output ($\Delta \kappa$) of the model using $L=50$ μm. (experimental data cf. Fig. 2B). (**J**) Moments ${M}_{na}(s,t)$ (each line represents moments at one time point along $s$). A few lines have been coloured green to demonstrate the node of vibration at 2nd bending mode. Comparable experimental data are in Fig. 3B.

Analysing model kinematic output in steady state conditions (using $L =10$ μm, $v =0.2$ m/s, $\alpha ~ = ~1$) showed that stick and slip phases and events were readily generated by the model. The choice of parameter values is explained in materials and methods (see Table 1 for a full list). Note that $v =0.2$ m/s corresponds to a shaft rotation of 420°/s. Base angular velocity (Fig. 5A) undershot driving velocity regularly and was followed by transients surpassing accelerations observed without contact. Searching for local extremes in these cases identified a stick–slip pattern similar to that seen in experiments (Fig. 5A,B) (cf. Fig. 1B). The model also captured the experimental fact that the whisker tended to vibrate at the second bending mode: tip and base accelerations were negatively correlated (Fig. 5B,C; correlation coefficient $r=-0.36$; the experimental data of comparable conditions [distance $l-1$ mm and smooth sandpaper surface, cf. Fig. 2B] yielded $r=-0.47$). Further, the experiment showed a tight correlation between kinematic and dynamical variables, a fact which the model captured: acceleration and normal force were correlated at a coefficient $r=0.44$. This correlation coefficient is lower than that seen in the experiment, perhaps reflecting that the model contact is with a smooth surface while the experiment used sandpaper surfaces containing asperities. Tip and base moment occur in opposite directions reflecting the second mode of bending (Fig. 5D,E,F, cf. Fig. 3C) (please note that the model used ${\kappa }_{i}=0$ for ease of computing [see materials and methods] explaining the shift of moment toward negative values, and therefore, a more moderate negative correlation [$r=-0.34$] as compared to the experiment). Plotting the average moment around stick events (Fig. 5G) we note that, as observed with the real whisker (cf. Figures 3E, 4C), stick events are followed by an increase in moment along the whisker. Thus, it appears that during a stick event, the moment builds up, and is subsequently released during the slip.

**Table 2:** List of all output parameters of the Cosserat and the friction model.

Kinematics	Symbol	Arguments
Angular position, velocity, acceleration	$\psi ,~v = \dot{\psi },~a = \dot{\psi }$	$s,t$
Curvature	$\kappa$	$s,t$
*Dynamics*		
Normal, axial force	$F_n, F_a (n\,and\, t\, in\, the\, model)$	$s,t$
Moment	${M}_{na}\,(M\,in\,the\,model)$	$s,t$
Friction	${F}_{T}$	$t$
Pressure	$p$	$s,t$

To adjust $L$ we ran the model varying $L$ (in the range from 5 to 500 μm). The resulting position spectra (Fig. 5H) revealed that the predominance of the second bending mode as observed in the experiment were best represented by middling values of $L$ (here 50 μm). Using the model with $L = 50\;\mu {\text{m}}$, to plot curvature $\Delta \kappa ={\kappa }_{i}-\kappa$ as done for experimental data in Fig. 2, yielded qualitatively similar oscillations to the ones observed with real whisker movement in contact with a smooth surface (Fig. 5I; cf. Fig. 2B). Calculating moments along the whisker beam revealed a strong increment from tip to base with the node of bending located in the lower half of whisker length (Fig. 5J), as seen in the experiment (Fig. 3B). As reported above, nodes (the signature of the second bending mode) were observed between 5 and 15 mm. The range of node locations are demonstrated by the four moment excursions highlighted in Fig. 5J.

## Discussion

This study provides fundamental insights about how vibrations are conveyed from tip to base of a rat conical whisker in moving contact with a texture. Biomechanical measurements reveal that conveyance of vibrations from tip to base is ultrafast, and utilizes the second mode of bending. Tip movements are transmitted to the base as moment, a dynamical variable. Due to tapering, the moment, which is vanishingly small at the tip, builds up to significant amounts at the follicle. A relative measure of forces acting on the follicle—their change in time—is available from kinematic data in the form of acceleration of the whisker beam close to the follicle. Stick–slip frictional movements feature prominently in dynamical signals conveyed down the beam. We demonstrate the novel mechanistic principles in a limited number of whiskers, such that the detailed intra and inter-individual variation of these phenomena has to be worked out in the future. We further established a realistic model of moving-contact whisker biomechanics based on Cosserat geometric theory linked to a state-rate friction law, which is the first to capture the rapid spatio-temporal transmission of frictional stick–slip movements along the whisker. This model qualitatively recreated the fast conduction, second order bending, and the conversion of frictional stick–slip movements at the whisker tip into robust bending moment at its base.

### Ultrafast transmission and second-order bending mode

The measurements of conveyance speed exceeded the temporal resolution of our ultrafast camera system (conveyance time from tip to base ($< ~0.25\;{\text{ms}}$). The conical shape of whiskers and the keratinous material suggest high pliability of the whisker, as has been previously observed^6^. This is reflected by our observation of a strong correlation between lateral deflection (${a}_{x}$) and normal force (${F}_{n}$). The inverse relationship of bending at tip and base was a surprising finding, and is explained by the robust observation that the static deformation of the whisker is in its second bending mode. Our mathematical model captured the phenomenon in relative terms (antagonistic relative movement) and partially in absolute terms (absolute direction of movement). We therefore conclude that the second bending mode is mechanistically brought about by the elastic and geometrical properties of the beam incorporated in the Cosserat model. A further role in specifying details of second bending mode vibration is played by the intrinsic curvature of the whisker. The typical movement direction in strongly whisking species (like the rat studied here) has been associated with distinct innervation patterns most conspicuously at the inner conical body of the follicle^35^. The observed inverse relationship of tip vs. base deflection (and amplified moment; see next paragraph) will likely be instrumental to decipher the mechano-electrical transduction in those specifically formed and distributed end organs.

### Transmission of frictional stick–slip movements to the follicle

Combining measurement of acceleration and force acting at the whisker base we found that the acceleration time series is a good proxy for the forces acting on the base: It is highly correlated with normal force ${F}_{n}$ and axial force ${F}_{a}$. This observation is likely to be of benefit for investigators, who work with behaving animals and who can thus only measure kinematic variables (positions, velocity and acceleration) but wish to infer dynamical variables (forces and moments) acting on the follicle. By direct measurement of acceleration, one could hope to capture at least the relative amplitude of dynamical variables. Absolute values of acceleration fluctuations at the whisker base, however, seem to vary significantly with the overall position of the node point of the second bending mode. A more robust measurement seems to be curvature fluctuations, and hence fluctuations of moment at the follicle. Indeed, we showed that the whisker appears to be an effective device for turning large distance, weak excursions of the pliable tip into short excursion, high moment fluctuations at the follicle.

The above-mentioned correlation of normal and axial forces, ${F}_{n}$ and ${F}_{a}$, acting on the whisker can be intuitively understood: Whenever normal force builds up—i.e. during stick phases—axial force (directed toward the tip) is increased because during a stick, the tip of the whisker stays behind and thus tends to pull out the base from its fixture. During slip the situation is roughly the inverse. In this study we were able to verify these intuitive relationships directly by assessing stick and slip events from videographic velocity and acceleration data^12^, with simultaneous assessment of moment (calculated from curvature, Fig. 3), and direct piezoresistive force measurements (Fig. 4). We found that moment ${M}_{na}$ and normal and axial forces $F_{n} ,$ build up during whisker stick events. In reverse, slip events are associated with the relaxation of these variables. Thus, we demonstrate that slips are robustly represented by dynamical variables along axial and normal dimensions. Experiments that found vivid responses of primary afferents after ramp-and-hold movements of the whisker along the two dimensions^36^ support the notion that the existent highly specialized classes of end organs^37^ partly have evolved to pick up follicle forces and moments along the two dimensions. The prominence of representations of stick–slip sequences in dynamic variables, shown here, suggests that it is those stick–slip events that dominate transduction into primary afferent action potentials. Prominent coding of temporally local deflection waveforms in primary afferents^14^, and the reported sparse but reliable response of S1 neurons to slips^16^ provide supporting evidence for this conjecture. As previous studies have concordantly shown that the kinematic profile of slips contains substantial texture information^10–12^, we hypothesize that the biomechanical transmission of stick–slip sequences, revealed here, sets the stage for neuronal coding of frictional movements and their hypothesized exploitation for purposes of texture discrimination^1^.

### Variation of biomechanical phenomena across whiskers and individuals

Mystacial whiskers are all long conical structures, the principle feature giving rise to the experimental data and modelling results reported here. In this initial study we focussed on the identification of new biomechanistic principles and did not attempt to give a detailed view about the variability of these principles. Slip transmission across whisker types on the snout, across animals, across the growth cycle of hair, and to different types of whiskers found in other species, are all topics left to be studied in future work. This said, we expect the core features reported here to generalize in some way across cone-shaped whiskers, as they are uniquely related to the tapering and pliability of the whisker tip. The fact that different whiskers (e.g. the small and immobile microvibrissae vs the mobile macrovibrissae) are used in different behavioural contexts^38^, imply that variations of slip coding might be found there. Specific behavioural functions of the well-described systematic changes in whisker morphology across the mystacial pad^5^ are unknown. The fact that encoding textures with slips varies systematically across the rat’s mystacial pad^12^, and whiskers of different locations show different frequencies of vibration when moving in free air^2,3^, point to a possible gradient in biomechanical mechanisms, also when in touch with a surface, that need to be aligned to the yet unknown functional aspects of whiskers ordered along arcs and rows.

### Mathematical model of whisker bending when in moving contact

We have established a mathematical model that, to our knowledge, is the first that exceeds quasi-static approaches as implemented before^6,23,24^, and uses an analytic mathematical formulation to capture critical dynamical features of whisker vibration under conditions of moving contact with a surface. Amongst them are the generation of stick–slip sequences and their transmission and translation into dynamical variables, the increment of moment along the beam, the second bending mode of vibration, and the correlation of kinematic variables with forces acting on the whisker base. The output of the model thus provides access to dynamical mechanical quantities (moments and forces), supposedly critical for tactile perception. We hold that the model will be well suited for future parametric studies towards understanding texture identification, as it implements two novel features that set it apart from previous approaches. Firstly, the model for the first time incorporates a frictional model, which we deem critical to study the sweeping touch across surfaces (which necessarily involves frictional contact), typically executed with the aim of texture identification. Secondly, the model is the first to use the Cosserat formulation of the whisker beam, which allows for arbitrarily large, geometrically exact deformations. With these two novel features in place, our model prepares the ground to model texture identification—without doubt an important function of whisker movements.

There are several aspects of the whisker vibrations described here that will benefit from non-linear geometry and friction model. The first is the ultra-rapid transmission of texture information from tip to follicle. Another particular aspect of tapered whiskers will surely require both novel aspects of the model: compared to hair of cylindrical shape (e.g. vellum hair), tapered whiskers rather quickly buckle when in moving contact to a surface. Such buckling will happen at the tapered parts close to the whisker’s tip, and will switch ‘point contact’ (only tip in contact with surface) to ‘line contact’ (contact made by a longitudinal stretch of the tapering whisker close to the tip). If, as appears highly likely, buckling is an issue in whisker related touch, both novel elements of our model, the frictional as well as the Cosserat sub-models, will be indispensable tools. In the present work, we simply followed the mathematical prediction that force is only transmitted from the lift-off point of the line contact. Thus, we assumed that a whisker with line contact over a length ${\boldsymbol{l}}_{0}$ is dynamically equivalent to whisker of length $\boldsymbol{l}-{\boldsymbol{l}}_{0}$ in point contact. While this may be correct at a first level of approximation, details in changing frictional forces when going from line to point contacts and vice versa may still turn out to be an important aspect in texture discrimination.

## Methods

### Kinematic measurement using videography

Moving contact in a constant context (i.e. fixed whisker speed and object distance) was established using a published protocol (Fig. 1A–C)^12^. In short, the whisker C4 was plucked from a dead animal (Sprague–Dawley, male, age 4 months), sacrificed for an experiment unrelated to the present report. Three whiskers (C4, C3, D4) were used in this study. Care was taken to secure the whisker in its entire length, that the follicle was included, and the hair shaft was devoid of kinks. Length, diameter and intrinsic curvature were measured using microscopic pictures before and after imaging the whisker. These measurements did not yield any measurable difference before and after the experiment. Confirming previous reports, the whisker was approximately of a conical shape^5,22^. The most salient divergence from a pure cone shape were shape irregularities on the last hundreds of microns of the tip. The cone’s tip was cut at the point where the beam measured 3 μm in diameter. The exact measurements of the whisker cone were: follicle length: 1056 μm; diameter at the base: 138.6 μm; tip diameter: 3.08 μm; length (s) from follicle to tip; 28.48 mm. Sandpapers of two grades were used: P80 (rough) and P1200 (smooth), both part of the standard series issued by the Federation of European Producers of Abrasives with mean grain diameters of 201 and 15.3 μm respectively. Free movement in air (contactless) of the whisker was also recorded for reference. Rotation speed was set to 420°∕s which is representative of a lower speed of whisking observed in vivo^39^. In some cases velocities of 840 and 1260°/s were used in addition (Fig. 3D,E). The whisker was clamped at its follicular site so that the whisker’s axis was perpendicular to the rotational axis of a shaft rotated by a stepper motor (Orientalmotor, Tokyo, Japan). A high-speed camera (GMCLTR1.3CL-SSF LTR Mikrotron, Unterschleissheim, Germany) with a Tokina objective (Tokina 100 mm f/2.8, AT-X PRO—Macro, 16 × 16 μm^2^/ pixel size, Kenko Tokina Co., Ltd., Japan) was positioned above the rotational plane of the whisker in order to record its planar motion. The acquired videos had a resolution of 480 × 270 pixels at 4 kHz (data in Fig. 3). Sandpapers were mounted on a cylindrical rigid plastic shield that was positioned in such a manner that its central axis coincided with the axis of the rotational shaft. Two different plastic shields were used, each precision-made using a 3D printer, having the geometry of cylindrical segments with radius 1 mm and 7 mm less than the whisker length (Fig. 1A). Hence the distance of the follicle to the textured surface can be varied, although it was held constant for the duration of a particular rotational movement. One measurement cycle consisted in forward movement of 60° across the sandpaper (convex surface of the whisker leading, as with protraction in the intact animal). The first 15 ms of trial time containing movement transients from rest to steady state were discarded.

### Force measurements

The force measurements (Fig. 4) were done using piezoresistive force sensors^31^, specially designed and fabricated for this purpose in the laboratory of authors I.S. and K.N. Design and the dimensions of the piezo-resistive force sensor is shown in figure S3. The sensor lever thickness and width was 20 μm, and 195 μm respectively. This type of sensor is sensitive to forces as small as 10 nN (see details in^31^). The whisker was mounted using micromanipulation on the lever using UV activated glue. The force sensor plus attached whisker were then carefully mounted on a central ledge, such that the whisker base was located in the centre of a rotational drum holding a sandpaper-clad cylindrical arena, identical to the one used for videography. The difference to the videography was that force sensor and vibrissa were held in place and the arena moved around them (sensor/vibrissa movement proved incompatible with the integrity of the sensor). Rotation velocity was 420°/s and distance of the arena to the whisker was 1 mm less than the whisker length. Thus, the relative speed and distance of movement of whisker across sandpaper was identical in experiments depicted in Figs. 1, 2, 3 and 4. For videography during force measurement a camera (Fastcom Mini WX100, Photron, Tokyo, Japan) at a resolution of 1600 × 360 pixels at 9.6 kHz was used (data in Fig. 4).

### Data analysis

We used *Matlab* to write a bespoke algorithm based on image contrast to track the whisker’s coordinates in the plane of its motion from each frame, leading to a time-series for a set of discrete points $\left\{ {x_{i} \left( t \right),~y_{i} \left( t \right)} \right\}$ along the whisker’s axis with the spatial resolution of 42 µm. Briefly, lighting was adjusted such that the acquired images were close to pure black and white with the white whisker on a black background. Starting from a manual starting point at the base the algorithm iteratively traced out the outline of the whisker by detecting the two steps in contrast (black to white and white to black) on a circle with radius of 154 µm around the last point that indicated the contours of the whisker. The centreline of the whisker was constructed as the centre between the contour steps and the starting point for the next iteration was chosen as end of the so-far constructed centreline (Fig. 1D shows a time sequence of centrelines). The resolution of the image was 14 × 14 µm. The data at each time-step was used to compute an arclength coordinate $s$ and the signed curvature at each material point along the whisker, respectively given by

4 $$s~ = \int_{{x_{0} }}^{x} {\sqrt {1~ + ~\left( {\frac{{dy}}{{dx}}} \right)^{2} } } ~dx,\quad ~\kappa ~ = ~x^{\prime } \left( s \right)y^{\prime\prime} \left( s \right) - ~y^{\prime } \left( s \right)x^{\prime\prime } \left( s \right),$$

where the prime refers to the derivative with respect to the arclength $s$, the derivatives being numerically evaluated by finite differences and the integral using the cumulative trapezoidal method. In keeping with simple estimation of axial stiffness, we make the assumption that the whisker is inextensible. Note that, as the spatial sampling frequency varied slightly from one frame to another (mostly due to loss of data points near the whisker’s tip), we used spline interpolation (via the *Matlab* function *interp1* to form a regular spaced grid of the arclength from the raw data, imposing an arclength increment of ds = 1 μm. The trajectory of any material point labelled by its arclength coordinate is then stored in an array of points $(x\left(s,t\right),y\left(s,t\right))$.

We also monitored the time series of the position angle $\psi \left( {s,t} \right): = \tan ^{{ - 1}} [y(s,t)/x(s,t)]$. The angular velocity is then $\dot{\psi }=\partial \psi (s,t)/\partial t$, as obtained from the numerical time derivative of $\psi (t)$. Subtracting the solid rotation of the whisker, from the imposed motion, we finally derive the rotation fluctuations kinematic variables

$$\stackrel{\sim }{\psi }=\psi -{\Omega }_{shaft}t$$ and $$\partial \stackrel{\sim }{\psi }/\partial t=\dot{\psi }-{\Omega }_{shaft}$$.

### Mathematical model of a clamped conical rod

The whisker is modelled as an inextensible elastic rod of length $l$ that is constrained to move in a plane, as depicted in Fig. S4. In figure S5 and related mathematical formulations, we present precise details of parameter, step sizes, boundary conditions needed to implement the model. The rod is assumed to have uniform material properties, but to have non-uniform cross-section representing the tapered nature of the whisker. The shape of the non-deflected beam corresponds to a truncated cone of cross-sectional area

$$A\left( s \right) = \pi r(s)^{2} ~,$$

and second moment of area given respectively by Eq. 1. Here $r$ is the whisker’s radius, which is assumed to vary linearly with the arclength coordinate $s\in \left[0,l\right],$ via

5 $$r\left( s \right) = r_{0} \left( {1 - \frac{s}{{l_{c} + l}}} \right)$$

where ${r}_{0}$ is the base radius and ${l}_{c}$ is the truncation length (i.e. the length of the tiny tapered whisker that would need to be added to make a perfect cone). This means the ratio of tip to base radius is $\frac{{r}_{1}}{{r}_{0}}=\frac{{l}_{c}}{{l}_{c}+l}$.

As is common in rod mechanics, we assume the rod to be unshearable and inextensible, and, in the absence of any evidence to the contrary, we suppose the rod to be linearly elastic (in an Euler–Bernoulli sense) with material properties that are uniform along its length. That is, the density $\rho$ and Young’s modulus $E$ are taken to be constant, with the only longitudinal variation of the corresponding linear density of mass $\rho A(s)$ and flexural rigidity $EI(s)$, due to the rod’s tapered geometry. Similarly, inspired by^40^ and to tame high frequency vibrations, we also include a geometrically spatially varying Kelvin-Voigt damping with coefficient $\delta$. With these assumptions, the mechanics of the whisker is described in terms of the local angle $\theta (s)$ between its centerline and the $x$ axis. It follows that the strain variable of the rod is measured in terms of its local curvature $\kappa \left( {s,t} \right) = ~\partial \theta \left( {s,t} \right)/\partial s.$ For whiskers, it may be assumed furthermore that the rod has intrinsic curvature, such that $\kappa ={\kappa }_{i}\left(s\right)$ in a load free configuration. As a result, only the moment (bending couple) constitutive equation is needed, which, in terms of the rod’s curvature $\kappa$ is written following^40^ as

6 $$M\left( {s,\kappa ,\dot{\kappa }} \right) = EI\left( s \right)\left[ {\kappa - \kappa _{i} \left( s \right)} \right] + \delta A\left( s \right)~\dot{\kappa }.~$$

For simplicity, all numerical results presented here were computed in the case ${\kappa }_{i}\left(s\right)$=0, which was found to make negligible difference to the transmission of dynamic information. This simplification can also be argued mathematically because rotary inertia is several orders of magnitude smaller than bending forces.

The rod was modelled to be in contact with a plate fixed at a finite distance $\stackrel{-}{y}<l$ from the base of the rod, and moving tangentially at a constant speed $v$ in the direction normal to the undeflected rod’s axis. Speed $v$ can be related to the angular velocity used in the experiments via the simple relation $v = ~\overline{y} \Omega _{{shaft}} .$ In all computations we assume tip contact, between the whisker and the plane. The normal pressure $p(t)$ at the tip is an unknown dynamical variable that is solved for as part of the problem. The base (follicle end) of the rod is assumed to be fixed within some rigid substrate representing the experimental shaft, leading to ideal clamped boundary conditions.

Under the above assumptions, the mathematical model is developed as follows. Material points of the rod are labelled by the Lagrangian coordinate $s\in [0,l]$ measured in the undeformed rectilinear configuration. The goal of the model is to describe the motion of each material point $r\left(s,t\right)=[x(s,t),y\left(s,t\right)]$ along the centreline of the rod. Let $f$ and $g$ be the Cartesian components of the force at each material point and let the corresponding moment be $m$. Under these hypotheses, conservation of linear momentum in each Cartesian direction and of angular momentum lead to the equations of motion

7 $$\rho A\left( s \right)\ddot{x} = f^{\prime},~\quad ~\rho A\left( s \right)\ddot{y} = g^{\prime},\quad \rho I\left( s \right)\ddot{\theta } = M^{\prime} + g~\cos \theta - f~\sin \theta,$$

in which partial derivatives with respect to time and arclength are denoted with a dot and a prime respectively. Inextensibility means that the forces $f$ and $g$ at each position $s$ are Lagrange multipliers, i.e. determined by kinematics not determined by constitutive laws. The inextensibility constraint requires that

8 $$x^{{\prime2}} + y^{{\prime2}} = 1~,$$

which is automatically satisfied by the differential equations

9 $$x^{\prime} = \cos ~\theta {\text{ }}~,~\quad y^{\prime} = \sin ~\theta ~.$$

Hence, with appropriate initial and boundary conditions, the Eq. 9 can be integrated to obtain the position of the rod $[x\left(s,t\right),y\left(s,t\right)]$ at each point along the rod and each instance in time. The base-end boundary conditions are straightforward. We suppose that this end is the origin of the Cartesian co-ordinates and that the rod is clamped, which gives

10 $$x\left( {0,t} \right) = y\left( {0,t} \right) = 0,~\quad ~~x^{\prime}\left( {0,t} \right) = 0,\quad ~~~\theta \left( {0,t} \right) = 0.$$

The boundary conditions at the tip end are less straightforward. The plate is assumed to occupy the half-space $y=\stackrel{-}{y}$ and be rigid. The tip is assumed to experience tangential and normal pressures $f_{T}$ and $p(t)$, which leads to boundary conditions of the form

11 $$y\left( {l,t} \right) = \overline{y} ,~\quad ~~~~f\left( {l,t} \right) = f_{T} ,~\quad ~~~~g\left( {l,t} \right) = - p,~~\quad ~~~~M\left( {l,t} \right) = 0.$$

Condition (Eq. 11, 1) when combined with (Eq. 9) leads to the integral constraint

12 $$\overline{y} = \smallint _{0}^{l} \sin ~\theta {\text{ }}~ds,~$$

which allows for determination of the unknown pressure $p(t)$.

### Friction model

A friction law was required to determine the tangential force ${f}_{T}(t)$ in terms of the pressure $p$ and the velocity $v$ of the plate’s motion. There is no single accurate model for frictional contact, especially in high-frequency dynamic environments, (see^32^). It is common to assume that the normal and tangential interfacial forces are related by a ‘coefficient’ of friction $\mu$ with a Coulomb or generalised Coulomb law between the slip velocity if the ratio of tangential to normal forces exceeds $\mu$. For solid interfaces, experimental evidence (see references in^33,41^) suggests that a match with experimental data may be obtained if $\mu$ is considered as a function of the past sliding history often modelled as an internal state variable that measures the state evolution of the frictional surface. Such so-called rate-and-state friction laws also have the advantage that they avoid the singularities associated with non-smooth Coulomb-like friction laws. Here we use a formulation^34^ in which we suppose $\mu$ to depend both on the velocity $v$ and a dimensionless, internal relaxation variable $\varphi \left(t\right)$, which measures the state of the interface and quantifies the interfacial resistance to slip.

Specifically

13 $$\left\{ {\begin{array}{*{20}l} {f_{T} = \mu \left( {\upsilon ,\varphi } \right)p~~~~~} \hfill \\ {\mu = F\left( {\upsilon ,\varphi } \right) = ~a~\sinh ^{{ - 1}} \left[ {\gamma _{*} \left( {\frac{v}{{V_{*} }}} \right)\varphi ^{{\frac{b}{a}}} } \right]~ \approx \mu _{*} + a\ln ~\left( {\frac{v}{{V_{*} }}} \right)~ + ~b\ln \left( \varphi \right);~\gamma _{*} \equiv \frac{{\exp \left( {\frac{{\mu _{*} }}{a}} \right)~}}{2}} \hfill \\ {\dot{\varphi } = - G\left( {\upsilon ,\varphi } \right) \approx - \frac{{\varphi - \varphi _{{ss}} \left( \upsilon \right)}}{{t_{{ \star }} (v)}};~~~~~\varphi _{{ss}} \left( \upsilon \right) \equiv \frac{{V_{*} }}{\upsilon };~~~~~t_{{ \star }} \left( \upsilon \right) \equiv \frac{L}{\upsilon }.} \hfill \\ \end{array} } \right.$$

The downside of the rate-and-state approach is that there are extra parameters that must be chosen (note that^33,41^ discuss ways for the experimental determination of such rate-and-state parameters). Specifically, ${\mu }_{*}\equiv \mu [{V}_{*},{\varphi }_{ss}({V}_{*})]$ is a kinetic coefficient of friction of reference and the parameters $a$ and $b$ respectively characterise the strength of the instantaneous velocity and state dependence. Note that, in the steady-state sliding situation, it is customary to distinguish between “velocity-strengthening” $(a-b>0)$ and “velocity-weakening” $\left(a-b<0\right)$ friction laws, the latter case allowing the possibility of stick–slip oscillations to exist. These parameters, as well as the parameter ${V}_{*}$, which is a characteristic reference velocity, can be associated to the microphysics of creep (see e.g.^34^ and references therein). The phenomenological sliding memory parameter $L$ represents a “slip-length” whose microscopic origin is still a matter of debate (see e.g.^32^ and references therein). The slip-length $L$ in the state evolution law (Eq. 13) models the characteristic length (or equivalently the timescale of order $L/v$) over which the frictional response to velocity jumps relaxes to a new sliding equilibrium^42,43^ and therefore we use L as a proxy for surface roughness.

We stress that the rate-and-state framework of friction is now well established for multi-contact interfaces over a wide range of scales. Nevertheless, dry friction modelling at the macro-scale based on microscopic measurements remains a topic of active research in the field of tribology, and further microscopic experimental work would however be needed to experimentally determine parameter regions of applicability of the rate-and-state formalism to whisker tribology.

### Numerical implementation and parameter fitting

The set of equations (Eq. 7) with constitutive equation (Eq. 6) and boundary conditions (Eqs. 10 and 11), associated with constraints (Eq. 9 and 12) constitute a well-posed system of algebraic partial differential system to be solved for the unknowns $\theta \left( {s,t} \right),~f\left( {s,t} \right),~g\left( {s,~t} \right)$ as well as the unknown scalars $p(t)$ and $\varphi (t)$.

The equations of motion of the whisker are numerically solved using the method of lines^48^. The discretization in space is achieved from the first order finite difference scheme proposed by^40^. The resulting large system of ordinary differential–algebraic equations of index 2 is then integrated in time using the implicit Runge–Kutta method of order 5 Radau IIA^44^ (see^45^ for a *Matlab* implementation*).*

With respect to parameter fitting, the whisker truncation length ${l}_{c}$ and density $\rho$ are readily calculated from the rod conical geometry that we assume. The value of Young’s modulus is based on the natural frequency of the second mode of vibration of the whisker that is exhibited from the power spectral density computed from the free-on-air data (see Fig. 2D). We tuned the value of $E$ from computing the frequency response of the whisker with a set of ringdown numerical experiments, i.e. the oscillatory response of the whisker whose tip has been displaced by applying a force. Our value is consistent with previous published estimates (e.g.^2,3,46,47^). The speed of sound ${c}_{s}=\sqrt{E/\rho }$ then follows directly. The driving plate location $\stackrel{-}{y}/l=0.98$ is chosen for the present geometry so that regional contact of the whisker tip is avoided, the tip contact remaining close to tangency. In absence of any tribological data for the system whisker/sandpaper, our choice of parameter values for the friction model is ad hoc and based on orders of magnitude commonly used in the relevant literature (see also Fig. S5 and related mathematical formulations).

## Supplementary Information

Supplementary Information.
