# Publisher Correction: Conveyance of texture signals along a rat whisker

**DOI:** 10.1038/s41598-021-03545-9

**Published:** 2021-12-14

**Authors:** Maysam Oladazimi, Thibaut Putelat, Robert Szalai, Kentaro Noda, Isao Shimoyama, Alan Champneys, Cornelius Schwarz

**Affiliations:** 1 Systems Neurophysiology, Werner Reichardt Centre for Integrative Neuroscience, University of Tübingen, Otfried Müller Str. 25, 72076 Tübingen, Germany; 2 Systems Neurophysiology, Hertie Institute for Clinical Brain Research, University of Tübingen, Tübingen, Germany; 3 Department of Engineering Mathematics, University of Bristol, Bristol, BS8 1UB UK; 4 Department of Sustainable Agriculture Sciences, Rothamsted Research, Harpenden, AL5 2JQ UK; 5 Department of Mechano‑Informatics, Graduate School of Information Science and Technology, University of Tokyo, Tokyo, Japan; 6 Department of Intelligent Robotics, Toyama Prefectural University, Toyama, Japan; 7 Toyama Prefectural University, Toyama, Japan

Correction to: *Scientific Reports* 10.1038/s41598-021-92770-3, published online 30 June 2021

The original version of this Article contained a repeated error in Equation 4 and 7 where an apostrophe was omitted from the variables “y”, “x”, and “M”.

As a result, in Equation 4,

$$s ={\int}_{{x}_{0}}^{x}\sqrt{1 + {\left(\frac{dy}{dx}\right)}^{2}} dx,\quad \kappa = {x}^{{\prime}}\left(s\right){y}^{{\prime}}\left(s\right)- {y}^{{\prime}}\left(s\right){x}^{{\prime}}\left(s\right),$$

now reads:

$$s ={\int }_{{x}_{0}}^{x}\sqrt{1 + {\left(\frac{dy}{dx}\right)}^{2}} dx,\quad \kappa = {x}^{{\prime}}\left(s\right){y}^{{{\prime\prime}}}\left(s\right)- {y}^{{\prime}}\left(s\right){x}{{^{\prime\prime}}}\left(s\right),$$

In Equation 7,

$$\rho A\left(s\right)\ddot{x}={f}^{{\prime}}, \quad \rho A\left(s\right)\ddot{y}={g}^{{\prime}},\quad \rho I\left(s\right)\ddot{\theta }=M+g\,cos\,\theta -f\,sin\,\theta ,$$

now reads:

$$\rho A\left(s\right)\ddot{x}={f}^{{\prime}},\quad \rho A\left(s\right)\ddot{y}={g}^{{\prime}},\quad \rho I\left(s\right)\ddot{\theta }={M}^{{\prime}}+g\,cos\,\theta -f\,sin\,\theta ,$$

The original article has been corrected.

