# Supplementary material for: Conveyance of texture signals along a rat whisker
Source: Sci Rep. 2021 Jun 30;11:13570. doi: 10.1038/s41598-021-92770-3 (PMC8245408; doi:10.1038/s41598-021-92770-3)
Supplement: Supplementary file 1 — Supplementary Information. [file 41598_2021_92770_MOESM1_ESM.pdf]

Supplementary Material (Figures S1, S2, S3, S4, S5, Supplemental text about the implementation of the model)

Conveyance of texture signals along a rat whisker Maysam Oladazimi, Thibaut Putelat, Robert Szalai, Kentaro Noda, Isao Shimoyama, Alan Champneys, Cornelius Schwarz

Fig. S1. Speed of transmission along the whisker beam. Moving contact at a distance $l-7$ mm **(A)** and $l-1$ mm **(B)**. In both panels the contact with the rough p80 sandpaper is shown at the top, contact with smooth p1200 sandpaper at the bottom. In the left columns acceleration traces measured at the tip (s=24mm) and base (s=3mm) are shown. The right columns show cross-correlograms of acceleration traces at tip vs. base, calculated from all trials measured in this configuration. The central negative peaks (vertical grey line) are located at time delay 0, up to measurement precision.

Fig. S2 Curvature data as shown in Fig. 2 but for smaller texture distance ($x = l - 7$ mm). With the whisker more engaged with the sandpapers, resonant phenomena are largely missing. A node with curvature change being zero is, however, preserved at 7-8 mm from the base. Arrows mark slip events that are transmitted from tip to base within one videography frame of 0.25 ms.

Fig. S3: Design and dimensions of the piezoresistive force sensor Four piezo-resistors are formed on to measure multi-axis forces applied to the tip of this cantilever type structure; two of them are formed on the surface of the beams as R1 and R4, and the others are formed on the side walls as R2 and R3. x and y directional forces applied to the tip of the sensor can be detected by comparing the resistance changes of these piezo-resistors formed on the spring type beams. The measured directional forces are then the and latitudinal (normal, x) and longitudinal (axial, y) forces acting on the whisker base (see figure 4 in the main text for orientation of the whisker mounted on the sensor).

Fig. S4: Cosserat Model. **A:** Conical whisker of length l. The model estimates the space-centroid line over time ($c(s,t) = [x(s,t), y(s,t)],\ \ with\ 0 < s < l$, green). At each point of c a cross-section circle exists ($radius\ r(s)$; a few circles are shown). The assumption of absence of shear along the beam guarantees orthogonality of circular cross-sectional planes to the tangent at $c(s,t)$. Proportions are not to scale (arbitrarily chosen to optimize visualization). **B:** The whisker is assumed to be in contact with a moving flat plate (violet, constant velocity v). The schematic shows the situation at one time point t. A characteristic variable of all points s is the angle θ between the horizontal axis and the tangent at $c(s)$, which defines the curvature $\kappa = d\theta/ds$. At each point $c(s)$ the acting force can be decomposed into axial and normal forces g and f, as well as normal and axial forces t and n (denoted F_a and F_n in Fig. 4; note the frame of reference of the modelling is different from the one used for measurements, cf. Fig. 4). The **inset** is an explanatory blow up from the site in the schematic marked by a grey rectangle. The time series of the angle ($\psi(c(s,t))$) provides a simple measure of movement of any point on c. The (instantaneous) dynamical friction coefficient μ is used to define the friction force $F_T = \mu\, p$, with pressure $p = p(l,t)$. F_T is determined by a 'rate and state friction' sub-model (see materials and methods for details).

Fig. S5: Model Implementation. Chain of rigid elements (μ) composing the model of the whisker beam. Index of elements: i; Number of elements: n; Forces: f, g; Moments: m. See supplementary text for more details.

Supplementary text related to figure S5.

Numerical implementation.

Here we present a full and condensed description of our Cosserat rod model of whiskers in dimensionless form, together with a finite difference semi-discretization scheme allowing its translation into a set of first order differential algebraic equations (DAEs) at the heart of the method of lines that we use to solve the equations of motion of the whisker.

The method of lines is a general technique for solving partial differential equations (e.g. see [48] for a didactic description of the method). It relies on the discretization of the spatial domain and derivatives which yields a large set of differential equations whose time integration can be performed using numerical routines developed for the numerical integration of ODEs and DAEs. Complete details are given to make the numerical implementation our method into a computer code in any computing language relatively straightforward.

We point out that our dimensionless formulation, explained below, renders our approach very generic in the sense that any whiskers with the same dimensionless characteristics will behave in exactly the same fashion. Another important feature of our formulation relies on the shape function $\sigma(s)$, which describes the rod geometry and its mechanical characteristics (like inertia and bending stiffness). The whisker geometry in this study is based on a linear cone, but any other shape could be assumed in principle from modifying σ to any other relevant and realistic function of the arclength s. With this view in mind, our formulation could be particularly useful for the study of vibrissae across the animal kingdom and for the design of synthetic whiskers to explore their mechanical response to dynamic contacts. Finally note that the present formulation is not restricted to a particular point contact model either.

All in all only slight modifications of the dimensionless formulation and its numerical implementation presented below would be required for the study of natural or synthetic whiskers with different shapes and with more complex inner structure.

Non-dimensionalisation and notations.

To non-dimensionalise system (7)–(13) in the main document and to resolve short wavelengths and high frequencies, we use $b/2$, $(b/2)/c$ and $\pi E b^2$ as characteristic scales of length (s) and displacement (x, y), time (t) and force (f, g), respectively. Note that the rod longitudinal wavespeed is denoted $c = \sqrt{E/\rho}$ and that moments are measured in units of $\pi E b^2 \ell$.

With abuse of notation, denoting the rod aspect ratio $\varepsilon = b/(2l)$, the dimensionless dynamics of the rod is governed by the partial differential-algebraic system,

$$\begin{cases} \sigma^2 \ddot{x} = f', \\ \sigma^2 \ddot{y} = g', \\ \sigma^4 \ddot{\theta} = (\sigma^4 \theta' + \delta \sigma^2 \dot{\theta}')' + g \cos\theta - f \sin\theta, \end{cases} \qquad \begin{cases} x' = \cos\theta, \\ y' = \sin\theta, \\ \bar{y}/\varepsilon = \int_0^{1/\varepsilon} \sin\theta \, ds. \end{cases} \tag{1}$$

The associated boundary conditions are straightforward to write down. The whisker shape function $\sigma(s)$ (i.e. dimensionless whisker radius) is defined as

$$\sigma(s) = (1 + \lambda_c - \varepsilon s)/(1 + \lambda_c), \tag{2}$$

for a truncated linear cone with a dimensionless truncation length $\lambda_c = \ell_c/\ell$ and a dimensionless arclength $s \in [0, 1/\varepsilon]$. Note that different whisker geometries could be considered from assuming different functions $\sigma(s)$.

The semi-discrete formulation of system (1) needed for the method of lines is obtained as follows. We have adapted the spatial finite difference discretization scheme of McMillen and Holmes (2006) [40] consisting in decomposing the rod over the discrete arc-length grid $s_i := ih$ ($i \in \{0, 1, \dots, n\}$), $h = 1/(n\varepsilon)$, as a chain of small rigid segments (labelled by index i) of length h,

mass μ_i and moment of inertia ν_i defined by

$$\mu_i := \sigma(ih)^2 h, \quad \nu_i := \sigma(ih)^4 h. \tag{3}$$

See figure S5. Correspondingly, the discrete version of the equations of motion reads

$$\mu_i \ddot{x}_i = f_{i+1} - f_i, \quad \mu_i \ddot{y}_i = g_{i+1} - g_i, \quad \nu_i \ddot{\theta}_i = m_{i+1} - m_i + n_i, \tag{4}$$

where the discrete shear force and moment are given by

$$n_i = (h/2)[(g_{i+1}+g_i)\cos\theta_i - (f_{i+1}+f_i)\sin\theta_i], \quad m_i = \sigma_{i-1}^4[(\theta_i - \theta_{i-1})/h - \kappa_{i-1}^0] + \delta\, \sigma_{i-1}^2(\dot{\theta}_i - \dot{\theta}_{i-1})/h. \tag{5}$$

The discretization of the constraint of inextensibility yields

$$x_{i+1} - x_i = (h/2)(\cos\theta_{i+1} + \cos\theta_i), \quad y_{i+1} - y_i = (h/2)(\sin\theta_{i+1} + \sin\theta_i), \tag{6}$$

while the quadrature formula that approximates integral conditions $(1)_6$ reads

$$\bar{y}/\varepsilon = h/2 + h\sum_{j=1}^{n} \sin\theta_j. \tag{7}$$

A discrete Differential Algebraic Equations (DAE) scheme.

We now express the system of equations above as a first order system of ODEs.

In the following, we denote $\mathbf{\Delta}_+ = \mathbf{E}_+ - \mathbf{I}$ the matrix corresponding to the difference operator $\Delta_+ f_i = f_{i+1} - f_i$, denoting the shift matrix $\mathbf{E}_+$ after the shift operator $E_+ f_i = f_{i+1}$. The vector $\mathbf{e}_i$ is the Euclidian basis vector that has unity in its i-th position and zeros elsewhere.

Hence, the discretization above translates into

$$\boldsymbol{\mu} \odot \ddot{\mathbf{x}} = \mathbf{\Delta}_+ \mathbf{f} + f_{n+1}\mathbf{e}_n, \quad \boldsymbol{\mu} \odot \ddot{\mathbf{y}} = \mathbf{\Delta}_+ \mathbf{g} + g_{n+1}\mathbf{e}_n, \quad \boldsymbol{\nu} \odot \ddot{\boldsymbol{\theta}} = \mathbf{\Delta}_+ \mathbf{m} + \mathbf{n} \tag{8}$$

where the symbol $\odot$ denotes the Hadamard (element-wise) product (i.e. $\mathbf{w} \odot \mathbf{v} = \mathrm{diag}(\mathbf{w})\mathbf{v}$). The discrete versions of the shear force and bending moment are given by

$$\begin{cases} \mathbf{n} = (h/2)[((\mathbf{E}_+ + \mathbf{I})\mathbf{g}) \odot \cos(\boldsymbol{\theta}) - ((\mathbf{E}_+ + \mathbf{I})\mathbf{f}) \odot \sin(\boldsymbol{\theta}) + g_{n+1}\cos(\theta_n)\mathbf{e}_n - f_{n+1}\sin(\theta_n)\mathbf{e}_n], \\ \mathbf{m} = (1/h)(\mathbf{E}_- \boldsymbol{\sigma}^4) \odot \mathbf{\Delta}_- \boldsymbol{\theta} - \theta_0 \mathbf{e}_1/h, \quad \theta_0 = \pi/2. \end{cases} \tag{9}$$

We denote $\boldsymbol{\sigma}^4$ the vector of component $(\boldsymbol{\sigma}^4)_i = \sigma(ih)^4 = \sigma_i^4$. The discretization scheme of the constraints $(1)_{4,5,6}$ gives the set algebraic constraints

$$\begin{cases} 0 = \mathbf{\Delta}_- \mathbf{x} - (h/2)(\mathbf{I} + \mathbf{E}_-)\cos(\boldsymbol{\theta}) - (h/2)\cos(\theta_0)\mathbf{e}_1, \\ 0 = \mathbf{\Delta}_- \mathbf{y} - (h/2)(\mathbf{I} + \mathbf{E}_-)\sin(\boldsymbol{\theta}) - (h/2)\sin(\theta_0)\mathbf{e}_1, \\ 0 = \bar{y} - \left[h/2 + h\,\mathrm{tr}(\mathrm{diag}(\sin(\boldsymbol{\theta})))\right], \end{cases} \tag{10}$$

necessary to solve the force vectors $\mathbf{f}$ and $\mathbf{g}$ and the tip pressure p. The symbols $\mathrm{tr}(\bullet)$ and $\mathrm{diag}(\bullet)$ respectively represent the trace of a matrix and the diagonal matrix made out of a vector argument.

Writing the vector of unknown variables

$$\mathbf{z} := (\mathbf{x}, \mathbf{y}, \boldsymbol{\theta}, \dot{\mathbf{x}}, \dot{\mathbf{y}}, \dot{\boldsymbol{\theta}}, \mathbf{f}, \mathbf{g}, p)^{\mathsf{T}}, \tag{11}$$

denoting for instance $\mathbf{x} = (x_1, \dots, x_n)^{\mathsf{T}} := (x(s_1), \dots, x(s_n))^{\mathsf{T}}$ ($(\bullet)^{\mathsf{T}}$ being the transpose of a vector), we then construct from (8)–(10) the DAE system to be solved

$$\mathbf{M}\dot{\mathbf{z}} = \mathbf{F}(\mathbf{z}) + \mathbf{F}_0, \tag{12}$$

by defining the mass matrix, the 'body' force and 'contact' vectors as

$$\mathbf{M} = \begin{pmatrix} \mathbf{I}_n & 0 & 0 & 0 & 0 & 0 & 0 & 0 & 0 \\ 0 & \mathbf{I}_n & 0 & 0 & 0 & 0 & 0 & 0 & 0 \\ 0 & 0 & \mathbf{I}_n & 0 & 0 & 0 & 0 & 0 & 0 \\ 0 & 0 & 0 & \mathrm{diag}(\boldsymbol{\mu}) & 0 & 0 & 0 & 0 & 0 \\ 0 & 0 & 0 & 0 & \mathrm{diag}(\boldsymbol{\mu}) & 0 & 0 & 0 & 0 \\ 0 & 0 & 0 & 0 & 0 & \mathrm{diag}(\boldsymbol{\nu}) & 0 & 0 & 0 \\ 0 & 0 & 0 & 0 & 0 & 0 & \mathbf{0}_n & 0 & 0 \\ 0 & 0 & 0 & 0 & 0 & 0 & 0 & \mathbf{0}_n & 0 \\ 0 & 0 & 0 & 0 & 0 & 0 & 0 & 0 & 0 \end{pmatrix}, \tag{13}$$

$$\mathbf{F}(\mathbf{z}) = \begin{pmatrix} \dot{\mathbf{x}} \\ \dot{\mathbf{y}} \\ \dot{\boldsymbol{\theta}} \\ \boldsymbol{\Delta}_+ \mathbf{f} + f_{n+1}\mathbf{e}_n \\ \boldsymbol{\Delta}_+ \mathbf{g} + g_{n+1}\mathbf{e}_n \\ \boldsymbol{\Delta}_+ \mathbf{m} + \mathbf{n} \\ \boldsymbol{\Delta}_- \mathbf{x} - (h/2)(\mathbf{I} + \mathbf{E}_-)\cos(\boldsymbol{\theta}) - (h/2)\cos(\theta_0)\mathbf{e}_1 \\ \boldsymbol{\Delta}_- \mathbf{y} - (h/2)(\mathbf{I} + \mathbf{E}_-)\sin(\boldsymbol{\theta}) - (h/2)\sin(\theta_0)\mathbf{e}_1 \\ \bar{y}/\varepsilon - \left[h/2 + h\,\mathrm{tr}(\mathrm{diag}(\sin(\boldsymbol{\theta})))\right] \end{pmatrix}, \quad \mathbf{F}_0 = \begin{pmatrix} \mathbf{0}_n \\ \mathbf{0}_n \\ \mathbf{0}_n \\ \mu(v_r, \varphi)\, p\, \mathbf{e}_n \\ -p\, \mathbf{e}_n \\ \mathbf{0}_n \\ \mathbf{0}_n \\ \mathbf{0}_n \\ 0 \end{pmatrix}. \tag{14}$$

We denote $\mathbf{I}_n$ the n-dimensional identity matrix, $\mathbf{0}_n$ representing either the zero matrix or vector of the relevant size.

When rate-and-state friction is assumed (see Eqs. (13) in the main document), this DAE system is augmented with the state evolution law equation (appropriately rescaled), the tip velocity relative to the driving surface (speed V) being evaluated with

$$v_r = V - [\dot{x}_n - (h/2)\sin(\theta_n)\dot{\theta}_n]. \tag{15}$$

Note that a change in the tip contact model would be implemented by modifying the non-zero components in the contact force $\mathbf{F}_0$.

In practice, the first order system of ODEs (12) was fed to the order 5 Radau IIA integrator [44] with automatic step-size control using the Matlab routine *radau5Mex* [45]. Note that the IDAS package for the solution of DAE systems from the SUNDIALS solvers [49] can be used as an alternative to the Radau integrator. The numerical results presented in the main document were computed with a spatial discretization based on a regular grid consisting of $n = 2^6$ grid points, the solution being evaluated in time at nodes equally spaced with $\Delta t = 1000$. Dimensionally, this corresponds to one material point every 0.44 mm with temporal snapshots every 22 μs.

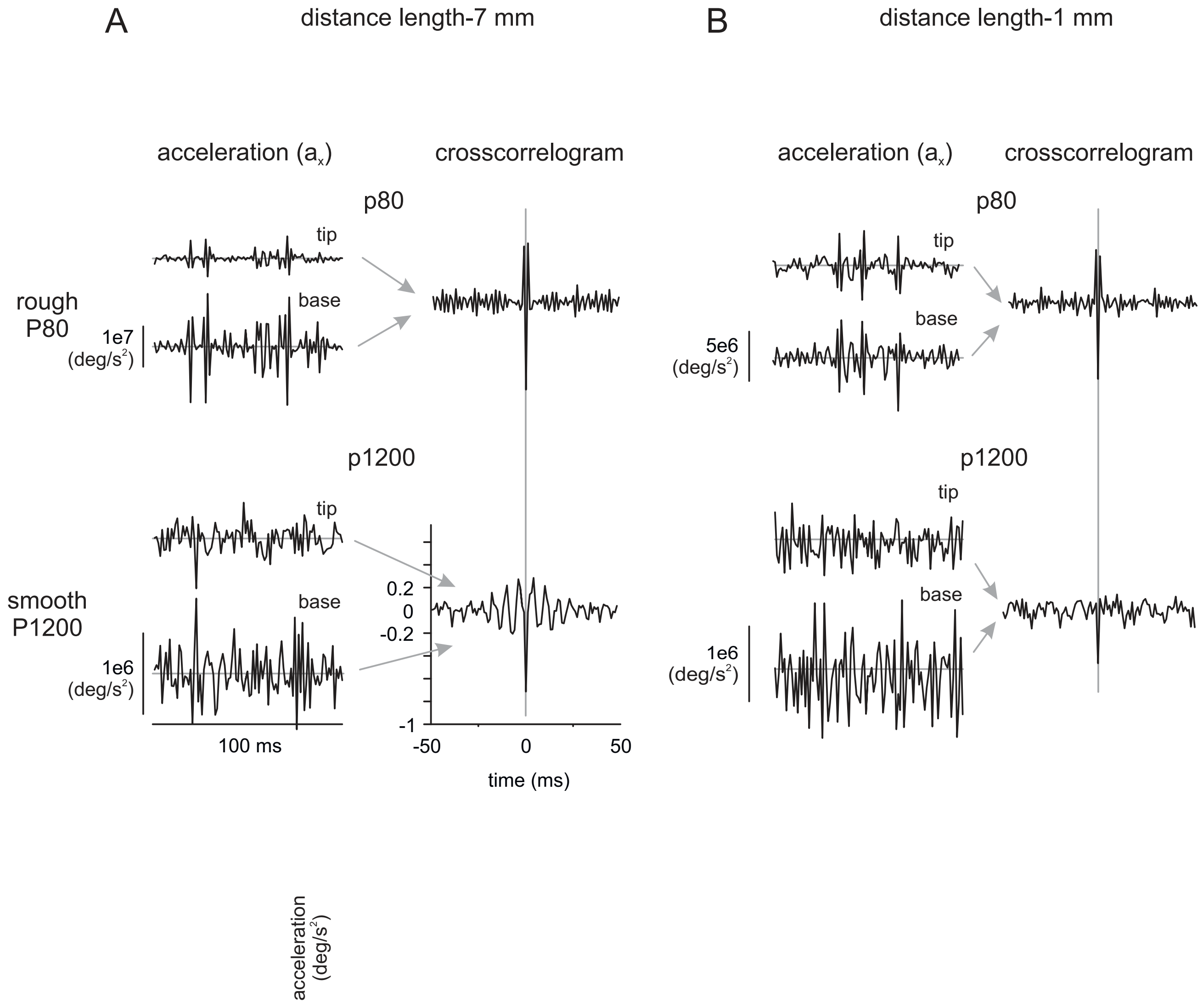

Oladazimi, Putelat et al.
Fig. S1

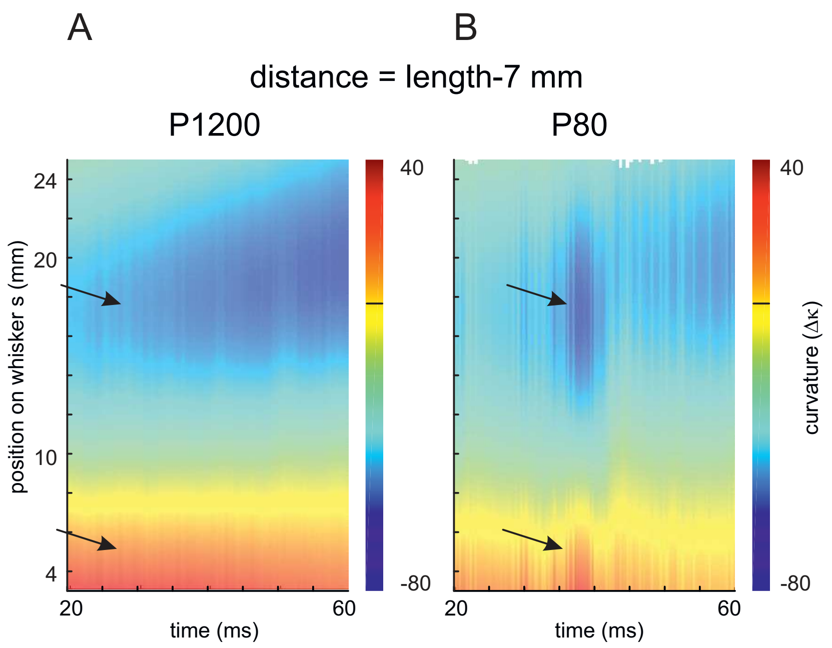

Oladazimi, Putelat et al.
Fig. S2

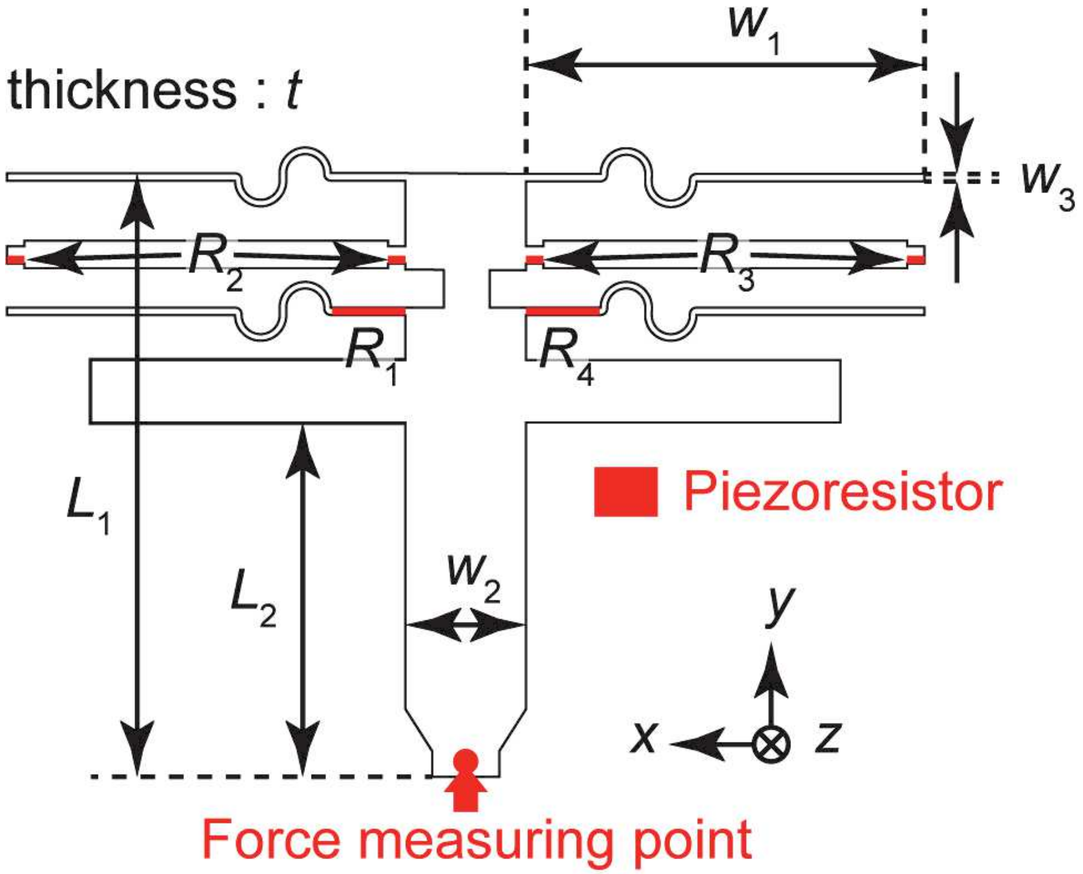

L_1	1800 μm	w_1	1200 μm
L_2	1065 μm	w_2	360 μm
t	35 μm	w_3	20 μm

Oladazimi, Putelat et al.
Fig. S3

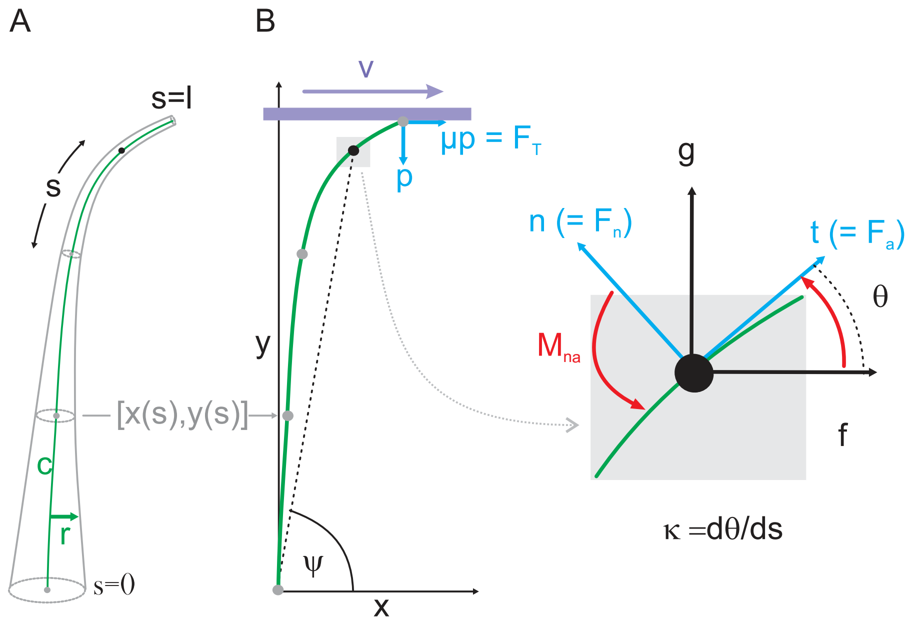

Oladazimi, Putelat et al.
Fig. S4

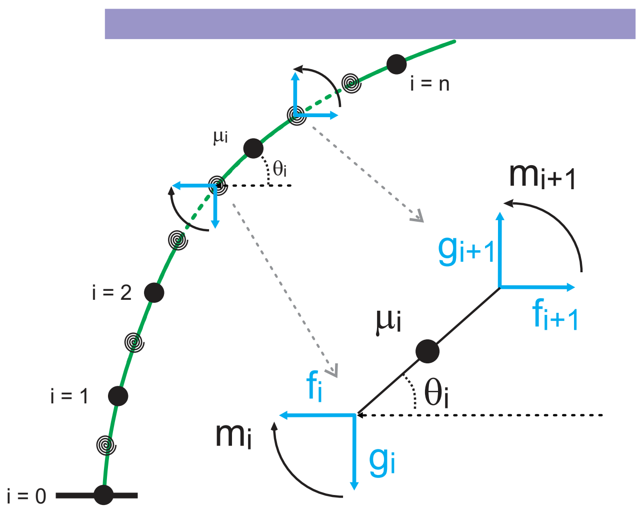

Oladazimi, Putelat et al.
Fig. S5
